# A score appraising Paleolithic diet and the risk of cardiovascular disease in a Mediterranean prospective cohort

**DOI:** 10.1007/s00394-021-02696-9

**Published:** 2021-10-21

**Authors:** Víctor de la O, Itziar Zazpe, Leticia Goni, Susana Santiago, Nerea Martín-Calvo, Maira Bes-Rastrollo, J. Alfredo Martínez, Miguel Á. Martínez-González, Miguel Ruiz-Canela

**Affiliations:** 1 Department of Preventive Medicine and Public Health, School of Medicine, University of Navarra, Irunlarrea 1, 31008 Pamplona, Spain; 2 Department of Nutrition, Food Sciences and Physiology, School of Pharmacy and Nutrition, University of Navarra, Pamplona, Spain; 3 CIBER Fisiopatología de la Obesidad y Nutrición (CIBEROBN), Instituto de Salud Carlos III (ISCIII), Madrid, Spain; 4 Navarra Institute for Health Research (IdiSNA), Pamplona, Spain; 5 Precision Nutrition Program, IMDEA Food, CEI UAM + CSIC, Madrid, Spain; 6 Department of Nutrition, Harvard TH Chan School of Public Health, Boston, USA

**Keywords:** Paleolithic diet, Mediterranean diet, Cardiovascular risk, Dietary patterns, Cohort study

## Abstract

**Purpose:**

To assess the association between a score appraising adherence to the PaleoDiet and the risk of cardiovascular disease (CVD) in a Mediterranean cohort.

**Methods:**

We included 18,210 participants from the *Seguimiento Universidad de Navarra* (SUN) cohort study. The PaleoDiet score comprised six food groups promoted within this diet (fruit, nuts, vegetables, eggs, meat and fish) and five food groups whose consumption is discouraged (cereals and grains, dairy products, legumes, culinary ingredients, and processed/ultra-processed foods). CVD was defined as acute myocardial infarction with or without ST elevation, non-fatal stroke and cardiovascular death. Cox proportional hazards models adjusted for potential confounders were fitted to assess the association between the PaleoDiet score and CVD risk, and the PaleoDiet and MedDiet indices to explore differences between both diets.

**Results:**

During 12.2 years of follow-up, 165 incident CVD cases were confirmed. A significant inverse association was found between the PaleoDiet score and CVD (HR Q5 vs. Q1: 0.45, 95% CI 0.27–0.76, *P* for trend = 0.007). A weaker association that became non-significant was observed when the item for low consumption of ultra-processed foods was removed from the score. Joint analysis of PaleoDiet and MedDiet Trichopoulou scores suggested that the inverse association between PaleoDiet and CVD was mainly present when adherence to the MedDiet was also high (HR for high adherence vs low adherence to both diet scores: 0.22, 95% CI 0.08–0.64).

**Conclusions:**

Our findings suggest that the PaleoDiet may have cardiovascular benefits in participants from a Mediterranean country. Avoidance of ultra-processed foods seems to play a key role in this inverse association.

**Supplementary Information:**

The online version contains supplementary material available at 10.1007/s00394-021-02696-9.

## Introduction

Cardiovascular disease (CVD) remains the most common cause of death, accounting for 2.2 million deaths in females and 1.9 million deaths in males during 2019 in Europe, and 31% of all deaths globally [1, 2]. Moreover, CVD incidence is globally increasing due to population growth, rural-to-urban migration and the aging of the world’s population [3]. CVD-related morbidity is associated with a gradual decrease in the quality of life and higher economic cost [4, 5], and it accounts for 24% of non-communicable diseases related disability-adjusted life years globally [5]. The promotion of healthy lifestyles to prevent CVD is an urgent need to reduce the current burden of this public health concern [6].

The promotion of healthy diets is probably one of the most cost-effective strategies to prevent CVD. Several dietary patterns have been proposed based on the cardioprotective effect of their components [7]. The Mediterranean Diet (MedDiet), the Alternative Healthy Eating Index or the Dietary Approaches to Stop Hypertension are well-known dietary patterns. They represent a priori defined high-quality diet scores inversely associated with the risk of CVD [8]. A common characteristic of these dietary patterns is a high consumption of fruits and vegetables, whole grains, nuts, legumes, vegetable oils, fish, and seafood; a moderate consumption of low-fat dairy products; and a low consumption of processed meat, sugar-sweetened beverages, and sodium [9].

Currently, there is an emergence of additional alternative dietary patterns with attributed health benefits although they usually rely only on limited scientific evidence. The Paleolithic diet (PaleoDiet) or “Diet Hunter-Gatherer” promotes the consumption of wild animal and plant food according to the supposed lifestyles of humankind during the Paleolithic age [10]. The PaleoDiet has increased its popularity during the last years [11], especially among young adults and athlete population, but also in patients with chronic diseases such as inflammatory bowel disease and celiac disease [12–14].

Anthropological studies have hypothesized that hunter-gatherers had a slim build, and they were fit and free of chronic diseases such as CVD due to their diet [15, 16]. From a public health perspective, the challenge is to know the potential health benefits of a Paleo-style dietary pattern adapted to current lifestyles and food availability. Currently, the PaleoDiet is characterized by a high consumption of fruits, vegetables, tree nuts, eggs, fish, and unprocessed meats, and a low consumption of dairy products, cereals and grains, legumes, processed foods, and culinary ingredients (added salt, sugar and refined fats) [17]. However, there is a diverse interpretation about the PaleoDiet at the popular levels and these Paleo-style diets may have no clear definitions and there is scarce scientific evidence to promote them [17]. Some recent reviews have suggested an inverse association between the PaleoDiet and cardiovascular risk factors [17, 18]. However, there is scarce evidence on the long-term beneficial effect of the PaleoDiet on hard end-points of the most prevalent chronic diseases such as CVD. To the best of our knowledge, only two studies with large sample size have suggested potential benefits of the PaleoDiet on CVD mortality [19, 20].

In this study we aimed to assess the association between a score appraising a PaleoDiet pattern and the risk of CVD in a well-known Spanish cohort. Knowing the association between this dietary pattern and CVD risk in a country with a relatively high adherence to the MedDiet is of interest. For this reason, we also explored the relationship between the MedDiet and PaleoDiet. While the MedDiet emphasizes a high consumption of all plant-based foods, the PaleoDiet limits the intake of legumes, cereals and grains, and recommends higher intake of unprocessed meats. Our hypothesis was that the PaleoDiet could reduce CVD risk due to the high consumption of fruits, vegetables, tree nuts, eggs, fish, unprocessed meats, and the exclusion of ultra-processed foods. In addition, we hypothesized that the recommendation to reduce the consumption of whole grains and legumes within the PaleoDiet score might mitigate the inverse association between this diet and CVD risk.

## Subjects and methods

### Study design and population

The *Seguimiento Universidad de Navarra* (SUN) study is a dynamic multipurpose prospective cohort of Spanish university graduates. A detailed description of the design and methodology is available elsewhere [21]. Briefly, self-reported questionnaires at baseline allowed to collect information about sociodemographic characteristics, lifestyle and eating habits, and health conditions of the participants. Information about some lifestyle factors and health outcomes has been updated biennially.

**Fig. 1:**
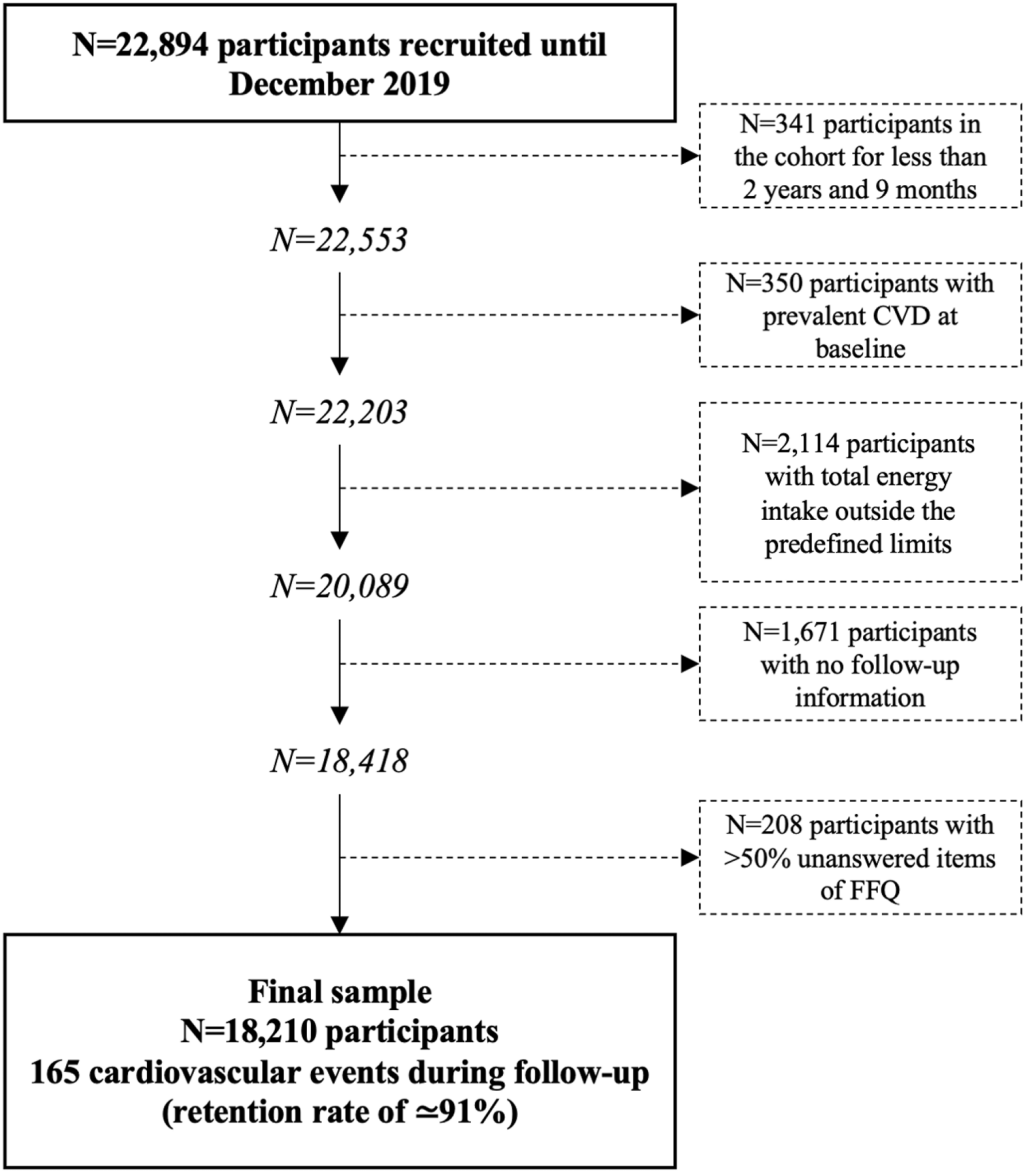
Flow-chart of participants recruited in the SUN Project, 1999–2019

The SUN cohort started in 1999 and since then, the total number of recruited participants until December 2019 was 22,894. From them, 22,553 participants were eligible for analyses of incident CVD to ensure that they could complete at least the first follow-up questionnaire. Additionally, we excluded 350 participants with prevalent CVD (myocardial infarction and revascularization), 2114 participants with total energy intake outside predefined limits (< 500 or > 3500 kcal/day for females, or < 800 or > 4000 kcal/day for males) [22], 1671 without follow-up information, and 208 with more than 50% of missing items in the semi-quantitative Food Frequency Questionnaire (FFQ). Therefore, 18,210 participants were included in our analyses (Fig. 1).

### Ethics

Participants received written information about the specific data required in the questionnaires, the protection to safeguard their privacy, and the future feedback from the research team. We also informed the potential candidates of their right to refuse to participate in the SUN study or to withdraw their consent to participate at any time without reprisal, according to the principles of the Declaration of Helsinki. The voluntary completion of the baseline questionnaire was considered to imply informed consent. The Research Ethics Committee of the University of Navarra approved this method to request the informed consent of participants. This cohort is registered at clinicaltrials.gov as NCT02669602.

### Dietary assessment

A validated 136-item semi-quantitative FFQ was used at baseline to collect information about diet [21, 23, 24]. Consumption frequencies were grouped into nine categories ranging from never/almost never to 6 + times per day. Total energy and nutrient intake were calculated using Spanish food composition tables [25, 26]. We multiplied typical portion sizes by consumption frequency for each food item to calculate the daily food consumption.

Ultra-processed foods were defined according to the classification system based on the extent and purpose of industrial food processing (NOVA food groups) [27]. Ultra-processed foods are defined as foods or drink products with low nutrient density and/or high energy density. The fourth NOVA category includes ultra-processed food and beverage that are typically created by series of industrial techniques and processes. Sugar, oils and fats, salt and additives that prolong product duration used to make processed foods, are often ingredients of ultra-processed foods, commonly in combination [27]. These products are usually ready for consumption such as carbonated drinks, sausages, biscuits and cookies, candies (confectionery), instant packaged soups and noodles, sweet or savory packaged snacks, and sugared milk and fruit drinks [27, 28]. To estimate the frequency of consumption of ultra-processed food we summed the amount consumed (servings per day) of each food item classified in the fourth category of the NOVA system (Supplemental Table 1). More detailed methodology has been described in previous studies conducted in the SUN cohort [28–30].

### The PaleoDiet score

The adherence to the PaleoDiet was assessed according to a published score that followed the theoretical definition of that dietary pattern according to previous definitions [17]. This index included six food groups promoted within the PaleoDiet (fruits, nuts, vegetables, eggs, meat and fish) and five food groups whose consumption is discouraged in this dietary model (cereals and grains, dairy products, legumes, culinary ingredients (added sugars, salt and refined fats) and processed/ultra-processed foods). We gave the same weight to all items and accordingly each one accounted for a maximum of five points in our score. All foods that comprised each group are shown in Supplemental Table 1. For each food group, participants were categorized into quintiles. Each quintile was assigned a numeric value ranging from 1 to 5 (an inverse punctuation was used for non-characteristic components of the PaleoDiet). The sum of all the components resulted in the PaleoDiet score with a theoretically minimum value of 11 and a maximum of 55.

### Assessment of Mediterranean Diet

We used two a priori defined MedDiet indices to assess the level of adherence to the traditional Mediterranean dietary pattern, which is characterized by a high intake of olive oil, vegetables, legumes, fruits, nuts, whole cereals, and fish; moderate consumption of red wine; low consumption of lean meat and dairy products, and very low or null consumption of red and processed meat. First, we used the Mediterranean Diet Score (MDS) developed by Trichopoulou and cols. (a widely used 9-unit scale based on nine dietary components that captures the essence of the traditional MedDiet) [31]. Second, we applied the 14-point Mediterranean Diet adherence Screener (MEDAS) used in the *Prevención con Dieta Mediterránea* (PREDIMED) trial, which includes five additional items that are also critical to an accurate assessment of the adherence to the traditional MedDiet, and is validated with objective biomarkers [32, 33]. Differences of these two MedDiet scores are briefly described in Supplemental Table 2.

### Outcome assessment

The endpoint was a composite of acute myocardial infarction with or without ST elevation, non-fatal stroke (both confirmed by a review of medical records with prior permission of relatives) and cardiovascular death. When a CVD was self-reported in a follow-up questionnaire, complete medical information was requested to the participant to confirm the CVD diagnosis by a cardiologist who was blind to diet and lifestyle exposure. In addition, we consulted the National Death Index annually to identify the deceased participants and to obtain their cause of death. In some cases, information about participants’ death was obtained from participants’ relatives, postal authorities and work associates.

### Statistical analysis

Baseline characteristics are described as mean ± standard deviation (SD) for quantitative variables and percentages for categorical variables. All variables shown in Tables 1 and 2 were age- and sex-adjusted using the Inverse Probability Weighting method [34]. Standard tests were applied to assess differences between the means and proportions of cohort characteristics according to PaleoDiet score quintiles.

**Table 1:** Age and sex-adjusted baseline characteristics of the participants of the SUN cohort according to quintiles of the PaleoDiet score (1999–2017)

	Quintiles of the Paleolithic diet score (min–max)				
	Q1	Q2	Q3	Q4	Q5
Score, min–max	(16–28)	(29–30)	(31–33)	(34–36)	(37–50)
*N*	4606	2699	4269	3515	3121
BMI, kg/m^2^	23.4 ± 3.6	23.5 ± 3.5	23.4 ± 3.4	23.7 ± 3.6	23.7 ± 3.6
Education level, years	5.1 ± 1.5	5.1 ± 1.5	5.1 ± 1.5	5.1 ± 1.5	5 ± 1.5
Smoking, %					
Non-smokers	47.5	48	49.3	48.8	49.7
Former smokers	27.1	27.3	28.8	30.4	30.5
Current smokers	24.6	24.1	21.2	20.1	18.9
Prevalent disease, %					
Hypertension	5.9	7.3	6.3	7.7	7.6
Hypertriglyceridemia	6.0	6.4	6.6	7.4	7.1
Hypercholesterolemia	16.8	17.2	16.9	16.9	17.0
Type 2 diabetes	0.8	1.3	1.7	1.9	2.9
Cancer	2.4	2.7	2.4	2.3	2.6
Depression	11.7	12.4	11.1	11.6	11.2
Napping, %	55.2	55.2	53.1	54.7	56.1
Snacking between meals, %	35.7	33.6	33.5	31.7	31.1
Special diets, %	4.8	6.0	7.2	10.1	13.9
Watching television, h/day	1.7 ± 1.2	1.6 ± 1.2	1.6 ± 1.2	1.6 ± 1.2	1.5 ± 1.1
Siting, h/day	5.3 ± 2.1	5.3 ± 2.0	5.3 ± 2.1	5.2 ± 2.0	5.2 ± 2.1
Physical activity, METs-h/week	18.7 ± 20.4	19.8 ± 21.8	22.1 ± 23.2	23.6 ± 23.3	26.6 ± 27.1
Mediterranean diet score (MDS) [31]	3.7 ± 1.6	4.0 ± 1.8	4.3 ± 1.8	4.7 ± 1.7	5.1 ± 1.6
Mediterranean Diet Adherence Screener (MEDAS) [32]	5.0 ± 1.6	5.6 ± 1.6	6.1 ± 1.7	6.6 ± 1.7	7.3 ± 1.7
Fruits, g/day	234.3 ± 215.6	297.4 ± 258.3	343.3 ± 282.3	408.5 ± 306.2	490.2 ± 337.4
Nuts, g/day	3.9 ± 6.7	5 ± 7.7	6.9 ± 10.8	9.3 ± 13.6	13 ± 16.3
Fish, g/day	70.2 ± 43.9	84.6 ± 48.6	97.7 ± 58.8	113.2 ± 62.6	135.1 ± 62.6
Eggs, g/day	21.1 ± 15.8	22.1 ± 14	23 ± 14	24.6 ± 16.7	26.5 ± 19.6
Vegetables, g/day	364.4 ± 230.1	463.2 ± 289.9	528.4 ± 319.6	623 ± 360.4	732.2 ± 371.6
Olive oil, g/day	17.5 ± 15.2	18.4 ± 14.6	18.6 ± 14.9	19.3 ± 14.8	19.6 ± 14.8
Unprocessed meats, g/day					
Lean meat	37.3 ± 31.4	41.3 ± 29.1	46.2 ± 33.1	51.3 ± 35.9	60.7 ± 42.5
Red meat	80.5 ± 44.6	89.2 ± 47.9	94.1 ± 50.6	98.1 ± 51.7	104.1 ± 56.7
Cereals and grains, g/day	119.2 ± 79.4	102 ± 70.8	94.3 ± 67.6	85.3 ± 66	70.3 ± 56.7
Dairy products, g/day	469.2 ± 262.7	423.3 ± 251.9	390.1 ± 247.2	368.2 ± 250.3	318 ± 244.3
Legumes, g/day	25 ± 18.8	22.7 ± 17.6	22.9 ± 17.5	22.3 ± 18.2	20.7 ± 17.2
Ultra-processed food, g/day	345.8 ± 204.8	314.5 ± 196.5	295.7 ± 190.6	280.8 ± 185.4	240.6 ± 159.3
Culinary ingredients, g/day					
Added salt	1.6 ± 1.4	1.6 ± 1.5	1.5 ± 1.4	1.5 ± 1.4	1.3 ± 1.4
Added sugar	19 ± 17.3	14.3 ± 15	11.2 ± 13.5	8.4 ± 11.8	5.6 ± 9.9
Refined fats	4.1 ± 7.9	3.1 ± 6.1	2.9 ± 6.6	2.4 ± 5.9	1.7 ± 4.9
Sugar-sweetened beverages, ml/day	56.4 ± 106.5	43.8 ± 78.9	38.1 ± 71.3	32.8 ± 73.3	20.8 ± 44

Means ± SD are shown unless otherwise stated. Table adjusted for age and sex by the Inverse Probability Weighting method

*BMI* body mass index, *MET* metabolic equivalent of task, *Q* quintile

**Table 2:** Age and sex-adjusted baseline nutritional values according to quintiles of the PaleoDiet score in participants of the SUN cohort (1999–2017)

	Quintiles of the Paleolithic diet score (min–max)				
	Q1	Q2	Q3	Q4	Q5
PaleoDiet Score, min–max	(16–28)	(29–30)	(31–33)	(34–36)	(37–50)
*N*	4606	2699	4269	3515	3121
Total energy, kcal/day	2421 ± 599	2349 ± 623	2331 ± 620	2343 ± 626	2311 ± 590
Macronutrients					
Carbohydrate intake, % of TEI	46 ± 70	44.1 ± 6.8	43.1 ± 7.1	42.1 ± 7.4	40.6 ± 7.6
Protein intake, % of TEI	16.6 ± 2.7	17.6 ± 2.7	18.3 ± 2.9	19 ± 3.2	20 ± 3.4
Animal protein	11.5 ± 3.0	12.4 ± 3.0	12.9 ± 3.2	13.5 ± 3.5	14.3 ± 3.7
Vegetal protein	5.2 ± 1.2	5.2 ± 1.2	5.4 ± 1.3	5.5 ± 1.3	5.7 ± 1.4
Fat intake, % of TEI	35.5 ± 6.5	36.2 ± 6.4	36.6 ± 6.5	36.9 ± 6.5	37.4 ± 6.6
SFA intake, % of TEI	12.6 ± 3.1	12.6 ± 3.1	12.5 ± 3.1	12.2 ± 3.2	12.0 ± 3.2
MUFA intake, % of TEI	14.9 ± 3.5	15.5 ± 3.5	15.8 ± 3.7	16 ± 3.7	16.4 ± 3.8
PUFA intake, % of TEI	5.1 ± 1.7	5.1 ± 1.5	5.1 ± 1.5	5.2 ± 1.4	5.3 ± 1.4
n-3 fatty acids, g/day	2.3 ± 1.3	2.4 ± 1.1	2.6 ± 1.2	2.8 ± 1.2	3.1 ± 1.2
n-6 fatty acids, g/day	19.8 ± 13.8	18.3 ± 12.2	17.3 ± 11.1	16.9 ± 10.6	15.8 ± 9.4
Fiber intake, g/day	23.5 ± 9.6	25.7 ± 11.1	27.8 ± 11.7	30.6 ± 12.7	34.2 ± 13.2
Alcohol intake, g/day	6.2 ± 9.4	6.7 ± 10.5	6.8 ± 10.8	6.6 ± 9.5	6.7 ± 10.2
Micronutrients					
Fe, mg/day	15.4 ± 4.3	16.2 ± 4.8	17 ± 5	18.1 ± 5.2	19.3 ± 5.3
Cr, µg/day	93.5 ± 41.7	87.7 ± 36.2	87 ± 36.3	86.1 ± 36.4	83.6 ± 32.3
I, µg/day	377.5 ± 219.5	346.8 ± 212.4	322.5 ± 192.6	313.9 ± 199.2	286 ± 183.7
Mg, mg/day	382.9 ± 105.4	396.2 ± 116.5	413.8 ± 122.2	438 ± 128	466.6 ± 129.4
Ca, mg/day	1243 ± 460.8	1222 ± 478.3	1219 ± 478.2	1234 ± 472.8	1221 ± 472.6
P, mg/day	1862 ± 501.9	1883 ± 521.9	1922 ± 545	1983 ± 550.9	2034 ± 551.4
K, mg/day	4148 ± 1242	4480 ± 1443	4755 ± 1525	5167 ± 1622	5623 ± 1659
Na, mg/day	3659 ± 2347	3402 ± 2179	3296 ± 2552	3198 ± 1970	2950 ± 1680
Na/K ratio	0.92 ± 0.61	0.79 ± 0.53	0.72 ± 0.53	0.64 ± 0.40	0.54 ± 0.32
Se, µg/day	89.9 ± 33.9	91.6 ± 32.6	95 ± 33.6	99.4 ± 35.3	105.2 ± 35.7
Zn, mg/day	16.4 ± 9.9	17.2 ± 10.2	17.9 ± 11.3	19.1 ± 11.2	19.7 ± 12.4
Vitamin A, µg/day	1405 ± 996	1747 ± 1357	1964 ± 1321	2294 ± 1593	2690 ± 1645
Vitamin B1, mg/day	1.7 ± 0.5	1.8 ± 0.5	1.8 ± 0.6	1.9 ± 0.6	20 ± 0.6
Vitamin B2, mg/day	2.2 ± 0.7	2.2 ± 0.7	2.2 ± 0.7	2.3 ± 0.7	2.3 ± 0.7
Vitamin B3, mg/day	37.6 ± 10.1	39.9 ± 10.7	42.2 ± 11.5	44.9 ± 11.9	48.6 ± 12.2
Vitamin B6, mg/day	2.3 ± 0.7	2.5 ± 0.8	2.8 ± 0.9	3.0 ± 0.9	3.4 ± 1.0
Vitamin B12, µg/day	7.9 ± 4.0	8.8 ± 4.3	9.5 ± 5.0	10.3 ± 4.9	11.7 ± 5.9
Vitamin C, mg/day	212.6 ± 110.6	253.9 ± 134.3	282 ± 146.7	318.9 ± 159.8	366.6 ± 174.3
Vitamin E, mg/day	6.5 ± 3.7	6.6 ± 3.3	6.8 ± 3.4	7.2 ± 3.5	7.8 ± 3.7
Folic acid, µg/day	331.2 ± 133.9	375.6 ± 156.9	410.9 ± 168.8	454.1 ± 176.8	512.3 ± 186.2

Means ± SD are shown unless otherwise stated. Table adjusted for age and sex by the Inverse Probability Weighting method

*MUFA* monounsaturated fatty acids, *PUFA* polyunsaturated fatty acids, *Q* quintile, *TEI* total energy intake, *SFAs* saturated fatty acids

We first analyzed the PaleoDiet score as a continuous variable and then categorized it into quintiles to define low (Q1), low-moderate (Q2), moderate (Q3), high-moderate (Q4) and high (Q5) adherence to the PaleoDiet. Cox proportional hazard regression models were used to estimate Hazard Ratios (HR) for CVD and their 95% confidence intervals (95% CI). We used the lowest quintile as the reference value and we also calculated the HRs for 5-unit increase in the PaleoDiet score.

In addition, we estimated the relative importance of each of the components of the PaleoDiet score by subtracting alternately one component from the original score, and afterwards estimating the HRs per 5-unit increment in the score. The comparability between the scores was preserved by multiplying the logarithm of the estimated HRs by 50/55 before exponentiating them (estimated HR for a 5-unit increment in PaleoDiet score = *e*(5 × [*β* coefficient of the PaleoDiet score without “item”]  ×  (50/55)) [35].

A crude model and three multivariable-adjusted models were fitted. Age was used as the underlying time-variable in all Cox models. In model 1 we adjusted for sex, and we stratified for age (deciles) and year entering the cohort (1999–2001, 2002–2004, 2005–2007, 2008–2010, 2011–2014, 2015–2017) using the option “strata” in STATA. In model 2 we additionally adjusted for total energy intake (continuous), alcohol intake (abstainer, > 0 to ≤ 5 g/day, > 5 to ≤ 25 g/day and > 25 g/day among females; abstainer, > 0 to ≤ 10 g/day, > 10 to ≤ 50 g/day and > 50 g/day among males), smoking status (non-smoker, former smoker, current smoker), pack-years of cigarette smoking (continuous), body mass index (BMI) (continuous), physical activity (METs-h/week as continuous), years of university education (continuous), family history of CVD (yes/no) and self-reported prevalent diseases at baseline, including hypertension, hypertriglyceridemia, hypercholesterolemia, diabetes, cancer, and depression. Finally, in model 3 we additionally adjusted for squared BMI, mid-day napping (yes/no), watching television (h/day), sitting time (h/week), between-meal snacking (yes/no) and following special diets (yes/no). To investigate linear trends across the quintiles of PaleoDiet scores we assigned the median value to each quintile and considered the variable as continuous.

We calculated Pearson correlation coefficients (*r*) to assess the strength and direction of the association between the PaleoDiet and the two MedDiet indices. To assess the potential effect of PaleoDiet in participants within a Mediterranean country, we conducted a joint analysis for the combination of MedDiet (using the MDS and MEDAS scores) and PaleoDiet scores. Both the MDS and PaleoDiet scores were divided into three categories (Q1, merged Q2–Q3–Q4, and Q5). Therefore, a joint variable with nine categories was created. The joint analysis for PaleoDiet and MEDAS were divided into three categories (Q1, merged Q2–Q3–Q4, and Q5) for PaleoDiet and into two categories according to the median for the MEDAS. Therefore, a joint variable with six categories was created. Radar plot is a useful technique for the graphic presentation of multivariate data [36]. We applied radar plots according to the joint analysis categories of PaleoDiet and MDS score to observe the standardized mean intake of the food groups used to define the PaleoDiet. The vertexes of the radar plot show the characteristic (dark light line) and non-characteristic (thick longitudinal line) foods of the PaleoDiet score.

Cox proportional hazard regression models were used to estimate the association between the joint categories of the PaleoDiet and MedDiet scores and CVD incidence, using the category with the lowest quintile for both MedDiet and PaleoDiet as the reference category.

We additionally conducted stratified analyses by sex, BMI (< 25 kg/m^2^ vs. ≥ 25 kg/m^2^), and leisure-time physical activity (≤ 20 METs-h/week vs. > 20 METs-h/week). Likelihood-ratio tests were applied to assess potential effect modification by these variables. We also conducted joint analysis for BMI (< 25 and ≥ 25 kg/m^2^) and physical activity (≤ 20 and > 20 METs-h per week) with adherence to the PaleoDiet score, again recategorized into 3 categories, Q1 (low adherence, as reference), merged Q2–Q3–Q4 (intermediate adherence), and Q5 (high adherence).

Finally, to assess the robustness of our findings we conducted the following sensitivity analyses: including self-reported but non-confirmed CVD events as incident cases; modifying limits of total energy < percentile 1 (P1) and > percentile 99 (P99) (i.e., excluding participants with total energy intake < 1073 or > 3777 kcal/day); excluding participants with a special diet at baseline; excluding participants < 40 years; excluding participants with chronic aspirin intake; excluding participants with hypertension at baseline; and additionally excluding participants with other causes of CVD different from the outcome prevalent at baseline (aneurysm of the aorta, heart failure, atrial fibrillation, pulmonary embolism, peripheral venous thrombosis and intermittent claudication).

We used STATA software to conduct all the analyses (STATA version 14.0, StataCorp, College Station, TX, USA). All *P* values presented are two-tailed. Statistical significance was considered at the conventional 0.05 level.

## Results

Among 18,210 participants (60.6% females), mean age at baseline was 38±12 years old. After a median follow-up of 12.2 years (209,867 person-years), we ascertained 165 incident cases of CVD (80 non-fatal myocardial infarctions, 60 non-fatal strokes, and 25 cardiovascular deaths).

Age- and sex-adjusted baseline characteristics of participants according to quintiles of the PaleoDiet score are summarized in Table 1. Differences in baseline characteristics according to quintiles of the PaleoDiet score were statistically significant (*P* < 0.001) except for education level (*P* = 0.763) and calcium intake (*P* = 0.106). Higher prevalent type 2 diabetes, and lower percentage of current smokers were observed across quintiles of the PaleoDiet score. Participants in the highest quintile practiced more physical activity, reported higher adherence to the MedDiet, and were more likely to follow special diets. Whereas, they were less likely to consume snacks between meals and watching television. As expected, participants across successive quintiles of the PaleoDiet score consumed more fruits, nuts, fish, eggs, vegetables, olive oil and unprocessed meats, and fewer cereals and grains, dairy products, legumes, ultra-processed foods, culinary ingredients and sugar-sweetened beverages.

Baseline age and sex-adjusted energy and nutrient intakes according to adherence to the PaleoDiet score are displayed at Table 2. Participants in the highest quintile referred the lowest total daily energy and carbohydrate intakes, but highest total protein (animal and plant source) and fat intake (monounsaturated (MUFA), polyunsaturated (PUFA) and n-3 fatty acids; and a lower intake of saturated (SFA) and n-6 fatty acids). As expected, participants with higher adherence to PaleoDiet consumed more fiber, Fe, K, Mg, P, Se, Zn, vitamin A, all vitamins from group B, vitamin C and vitamin E than those with lower adherence. In addition, a lower Na/K ratio was observed in the higher quintiles of the PaleoDiet score.

Table 3 shows a significant inverse association between the highest quintile of the PaleoDiet score and CVD risk compared to the lowest quintile in all regression models. A significant inverse association was also found for Q3 but not for Q4, although the *P* for linear trend was statistically significant in all models. A 19% (95% CI 32–4%) reduced CVD risk was observed for each 5-unit increment of the PaleoDiet score (model 3).

**Table 3:** Relative risk of cardiovascular disease (hazard ratios) according to quintiles of the PaleoDiet score among participants of the SUN cohort (1999–2017)

	Quintiles of Paleolithic diet score (min–max)						HR (95% CI)
	(Q1)	(Q2)	(Q3)	(Q4)	(Q5)	*P* trend^a^	
Limits	16–28	29–30	31–33	34–36	37–50		Per 5-unit increment
Cases/person-y	42/55,306	22/31,460	31/49,231	42/39,941	28/33,985		
Crude	1 (Ref.)	0.69 (0.41, 1.16)	**0.50 (0.31, 0.80)**	0.65 (0.42, 1.01)	**0.42 (0.26, 0.68)**	0.001	**0.78 (0.66–0.93)**
Model 1	1 (Ref.)	0.72 (0.43, 1.23)	**0.50 (0.31, 0.81)**	0.72 (0.46, 1.13)	**0.47 (0.28, 0.78)**	0.008	**0.81 (0.68–0.96)**
Model 2	1 (Ref.)	0.69 (0.40, 1.19)	**0.50 (0.30, 0.82)**	0.70 (0.44, 1.12)	**0.44 (0.26, 0.73)**	0.005	**0.80 (0.67–0.95)**
Model 3	1 (Ref.)	0.69 (0.40, 1.19)	**0.51 (0.31, 0.83)**	0.72 (0.45, 1.15)	**0.45 (0.27, 0.76)**	0.007	**0.81 (0.68–0.96)**

Crude model adjusted for age (10 groups) as the underlying variable of time. Model 1 adjusted for sex, stratified for age deciles and year entering the cohort (1999–2001, 2002–2004, 2005–2007, 2008–2010, 2011–2014, 2015–2017). Model 2 additionally adjusted for total energy intake (continuous), alcohol intake (teetotaler, > 0–5 g/day in females and > 0–10 g/day in males, > 5–25 g/day in females and > 10–50 g/d in males, > 25 g/day in females and > 50 g/day in males), smoking status (non-smoker, ex-smoker, current smoker), BMI (continuous), physical activity (METs-h/week as continuous), prevalent hypertension, hypertriglyceridemia, hypercholesterolemia, diabetes, cancer, depression and family history of CVD (yes/no), education level (graduate, master, doctorate) and smoking-pack-years (continuous). Model 3 additionally adjusted for squared BMI, napping (yes/no), watching television (h/day), sitting time (h/week), snacking between meals (yes/no) and following special diets (yes/no)

Bold HR and 95% CI reflects a significant result (*P *< 0.05)

*HR* hazard ratio, *CI* confidence interval, *Q* quintile, *Ref.* reference

^a^Test for lineal trend calculated for the 5 quintiles

We repeated Cox regression analysis after alternatively excluding one dietary component of the PaleoDiet score at a time (Table 4). No substantial changes were observed for these HRs compared to the HR calculated for the original PaleoDiet score except when the contribution of ultra-processed foods (as continuous variable per 5-unit increment), and when ultra-processed foods, fruits and vegetables (comparison between extreme quintiles) were removed from the PaleoDiet score the inverse association between the PaleoDiet score and CVD was reduced, and the *P* value was no longer statistically significant. Moreover, significant but weaker inverse associations were found for the alternative PaleoDiet scores without nuts or fish, and stronger association when cereal and grains did not negatively score (Table 4).

**Table 4:** Hazard ratios and confidence intervals after alternate subtraction of each of its dietary components among participants of the SUN cohort

	HR^a^ (95% CI) per 5-unit increment	HR^b^ (95% CI) Q5 vs. Q1 (Ref.)	*P* for trend^c^
Overall	0.81 (0.68–0.96)*	0.45 (0.27, 0.76)**	0.007
Excluding one item with a positive punctuation in the PaleoDiet score			
PaleoDiet score without eggs	0.81 (0.68–0.97)*	0.41 (0.24–0.70)**	0.008
PaleoDiet score without vegetables	0.82 (0.67–0.99)*	0.62 (0.36–1.09)	0.080
PaleoDiet score without fruits	0.83 (0.69–1.00)*	0.67 (0.39–1.15)	0.104
PaleoDiet score without nuts	0.79 (0.66–0.96)*	0.55 (0.31–0.96)*	0.032
PaleoDiet score without fish	0.81 (0.67–0.98)*	0.54 (0.32–0.91)*	0.011
PaleoDiet score without unprocessed meats	0.80 (0.66–0.69)*	0.47 (0.28–0.79)**	0.009
Excluding one item with a negative punctuation in the PaleoDiet score			
PaleoDiet score without cereals and grains	0.76 (0.63–0.91)**	0.40 (0.23–0.68)**	0.001
PaleoDiet score without dairy products	0.82 (0.68–0.98)*	0.53 (0.31–0.90)*	0.030
PaleoDiet score without legumes	0.81 (0.68–0.96)*	0.47 (0.28–0.78)**	0.011
PaleoDiet score without ultra-processed foods	0.85 (0.71–1.02)	0.64 (0.38–1.08)	0.131
PaleoDiet score without culinary ingredients	0.78 (0.64–0.94)**	0.53 (0.31–0.90)*	0.010

*HR* hazard ratio, *CI* confidence interval, *Q* quintile, *Ref.* reference

*P < 0.05

**P < 0.01

^a^Originally estimated logarithms of hazard ratios were multiplied by 50/55 and then exponentiated to correct for 50-point scale

^b^Hazard ratio adjusted for age (10 groups) as the underlying variable of time, sex, year entering the cohort (1999–2001, 2002–2004, 2005–2007, 2008–2010, 2011–2014, 2015–2017), total energy intake (continuous), alcohol intake (teetotaler, > 0–5 g/day in females and > 0–10 g/day in males, > 5–25 g/day in females and > 10–50 g/day in males, > 25 g/day in females and > 50 g/day in males), smoking status (non-smoker, ex-smoker, current smoker), BMI (continuous), physical activity (METs-h/week as continuous), prevalent hypertension, hypertriglyceridemia, hypercholesterolemia, diabetes, cancer, depression and family history of CVD (yes/no), education level (graduate, master, doctorate) and smoking-pack-years (continuous), squared BMI, napping (yes/no), watching television (h/ day), sitting time (h/week), snacking between meals (yes/no) and following special diets (yes/no)

Bold HR and 95% CI reflects a significant result (*P* < 0.05)

^c^Test for lineal trend calculated for the five quintiles

Subgroup analysis suggested no relevant differences according to levels of leisure-time physical activity, sex, or BMI (*P* for interaction = 0.730, 0.616 and 0.180, respectively) (Supplemental Fig. 1).

**Fig. 2:**
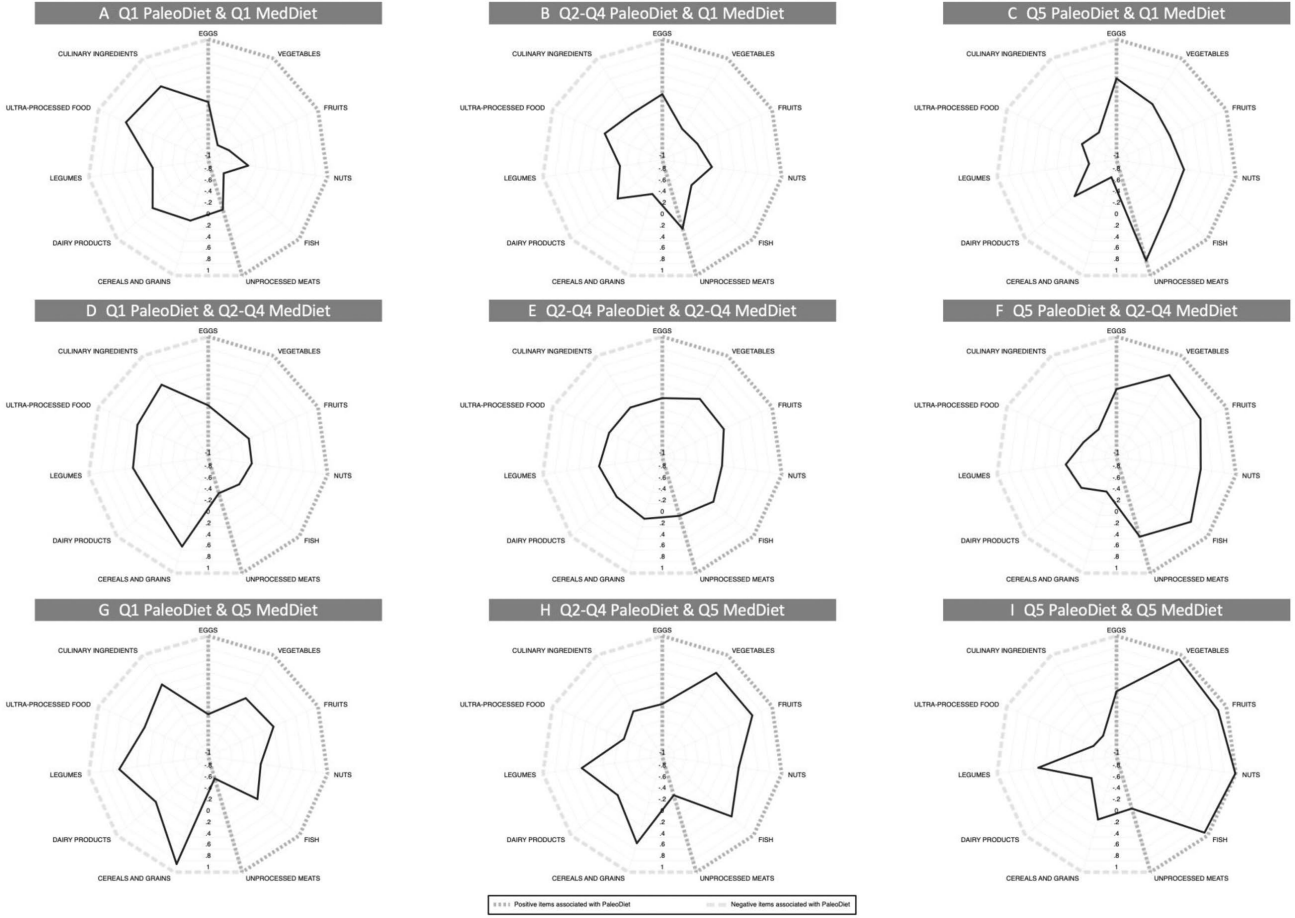
Mean intake serv/day (per SDs) of common group foods in both patterns according to the categories of joint analyses of the recategorized PaleoDiet and MedDiet scores (MDS, (28)). *C* Category. The radar plot axis is expressed in standard deviations (SD). 1-SD of eggs is equivalent to 1.5 serv/day; 1-SD of vegetables is equivalent to 1.6 serv/day; 1-SD of fruits is equivalent to 1.2 serv/day; 1-SD of nuts is equivalent to 0.6 serv/day; 1-SD of fish is equivalent to 1.7 serv/day; 1-SD of unprocessed meats is equivalent to 2.1 serv/day; 1-SD of cereals and grains is equivalent to 1.4 serv/day; 1-SD of dairy products is equivalent to 1.7 serv/day; 1-SD of legumes is equivalent to 1.3 serv/day; 1-SD of ultra-processed foods is equivalent to 1.9 serv/day; 1-SD of culinary ingredients is equivalent to 2.1 serv/day

Pearson correlation coefficients between the PaleoDiet score and MDS and MEDAS, were 0.32 and 0.48, respectively. Figure 2 shows the standardized mean intake (servings/day) of the food groups used to create the PaleoDiet score in the nine categories of the joint combination of categories according to three groups of the PaleoDiet score and three groups of the MedDiet (MDS) score. The axis of the radar plot represents the mean consumption per day of each food group in units of SDs, being − 0.8 the minimum value and + 1 the maximum value. Figure 2c, f and i show that among participants with the highest adherence to the PaleoDiet (Q5), all positive items associated with the Paleodiet, but also legumes and cereals and grains, increased with a higher adherence to the MedDiet. Moreover, Table 5 shows that the strongest inverse association with CVD risk was observed among participants with the highest quintile for both the PaleoDiet and the MedDiet scores (*P* for trend < 0.001). When we applied the joint analysis of the PaleoDiet and the MEDAS scores, we also observed an inverse association with CVD risk among participants with the highest quintile for both scores compared to participants with the lowest adherence to both diets. (HR 0.51; 95% CI 0.28, 0.92; *P* for trend = 0.004) (Supplemental Table 3).

**Table 5:** Hazard ratios and confidence intervals according to joint classification by combined exposures to the PaleoDiet and the MedDiet score (MDS)

	Paleolithic diet score (min–max)		
	Q1 (16–28)	Q2–Q4 (29–36)	Q5 (37–50)
Mediterranean diet score (min–max)			
Q1 (0–3)	1 (Ref.)	0.64 (0.34–1.20)	0.52 (0.17–1.53)
Q2–Q4 (4–6)	0.73 (0.38–1.42)	**0.48 (0.26–0.86)**	**0.36 (0.18–0.74)**
Q5 (7–9)	0.21 (0.03–1.61)	**0.28 (0.12–0.62)**	**0.22 (0.08–0.64)**

MedDiet score (MDS) was assessed using the Trichopoulou’s score [31]

Hazard ratio adjusted for age (10 groups) as the underlying variable of time, sex, year entering the cohort (1999–2001, 2002–2004, 2005–2007, 2008–2010, 2011–2014, 2015–2017), total energy intake (continuous), alcohol intake (teetotaler, > 0–5 g/d in females and > 0–10 g/day in males, > 5–25 g/day in females and > 10–50 g/day in males, > 25 g/day in females and > 50 g/day in males), smoking status (non-smoker, ex-smoker, current smoker), BMI (continuous), physical activity (METs-h/week as continuous), prevalent hypertension, hypertriglyceridemia, hypercholesterolemia, diabetes, cancer, depression and family history of CVD (yes/no), education level (graduate, master, doctorate) and smoking-pack-years (continuous), squared BMI, napping (yes/no), watching television (h/day), sitting time (h/week), snacking between meals (yes/no) and following special diets (yes/no)

Bold HR and 95% CI reflects a significant result (*P* < 0.05)

*Q* quintile, *Ref.* reference

Lastly, results from sensitivity analyses did not substantially change except when we applied different total energy intake limits to exclude participants (< P1 and > P99, total energy intake < 1073 or > 3777 kcal) and when we excluded participants younger than 40 years (Supplemental Table 4). In these two cases, CVD risk was reduced in 65% and 43%, respectively, in the fifth quintile as compared to the lowest quintile.

## Discussion

This prospective Mediterranean cohort study, conducted among young adult participants, showed an inverse association between higher adherence to the PaleoDiet and CVD risk. Among participants with the highest PaleoDiet score (upper quintile), a 55% relative CVD risk reduction was observed as compared to participants in the lowest quintile. This association was not significantly modified by sex, weight status or physical activity, and the results were robust in multiple sensitivity analyses aimed at controlling for residual confounding. A similar association was also observed when we alternatively excluded items one by one from the PaleoDiet score, although the consumption of fruits and vegetables, and the avoidance of ultra-processed foods may be key components of this diet. However, weaker inverse associations were also found when nuts or fish were excluded, suggesting the synergistic effect of all components within the PaleoDiet score. However, the prohibition of grains and cereals should be further explored since a stronger inverse association was found when this limitation was not part of the PaleoDiet score. Finally, in the joint analysis according to levels of adherence to PaleoDiet and MedDiet, the strongest inverse association with CVD was found among participants with the highest adherence to both dietary patterns.

Our findings are consistent with those published in a previous cohort study supporting the inverse association between the PaleoDiet and CVD death. In the REGARDS cohort study, with 21,423 participants and 863 CVD deaths, cardiovascular death risk was reduced by 22% in participants who most adhered to the PaleoDiet score (Q5) compared to the lowest quartile, although a borderline significance was observed [19]. In this study, only a stronger inverse association was found for the MedDiet compared to the PaleoDiet for total and specific causes of death. The Moli-sani cohort study, which used a similar PaleoDiet definition as Whalen and cols. [37], found a significant association between the PaleoDiet and total death but no significant association with cardiovascular mortality [20]. In that study, no modification effect of the PaleoDiet was observed in a stratified analysis by level of adherence to the MedDiet [20]. In another study, a score comprising dietary habits and other lifestyle behaviors that could be concordant with a PaleoDiet lifestyle such as limited alcohol consumption, not smoking, high levels of physical activity, and low levels of sedentary behavior showed an inverse relationship with all CVD mortality [38].

The inverse association between the PaleoDiet and CVD risk is supported by the previously reported effect of the PaleoDiet on different markers of CVD risk [18]. The PaleoDiet has shown a significant reduction in the risk of anthropometric markers such as body weight [12, 39–43], waist circumference [12, 39–42], BMI [12, 40, 41], and the percentage of fat mass [39, 42, 44]. Additionally, those who followed a PaleoDiet-style lowered their systolic and diastolic blood pressure, total blood cholesterol, triglycerides, LDL-cholesterol and had increased levels of HDL-cholesterol [12, 39–43]. However, most of these studies had a small sample size and short follow-up [17].

The potential beneficial effects of the PaleoDiet for cardiovascular risk could be attributed to a high consumption of fruits, vegetables (important sources of fiber), fish and nuts, MUFAs and PUFAs, as well as to a limited consumption of ultra-processed foods, added sugar, salt and refined oils intake. Similar to traditional and ancient diets, the PaleoDiet promotes higher nutrient density [45] and higher fiber intake [46] compared to current Western diets [47]. In comparison with Western diets, in a previous study it was observed that PaleoDiet could contain 200–300% more fiber, 150–200% more PUFAs and MUFAs, 400% more omega-3 fatty acids, but 60–70% less SFAs [15].

Eaton and cols. estimated a high consumption of vegetable foods in Paleolithic diets and, according to this, the preagricultural fiber intake exceeded 100 g/day [46, 48]. Fiber consumed by humans during the Paleolithic area came primarily from fruits, legumes, nuts, and other noncereal vegetable sources, and its content of phytic acid would had been less than that of the fiber consumed now in industrialized nations, which comes largely from grains [48]. According to Eaton and Konner, the proportion of soluble, fermentable fiber relative to insoluble, non-fermentable fiber was likely higher in meals consumed by preagricultural humans [48]. In the current study, we only found 50% more fiber in the lowest vs. highest PaleoDiet quintiles and there was only a small difference in SFA intake. The low mean SFAs intake found in our study is explained because the PaleoDiet score quintiles depend on the distribution of SFAs in our Mediterranean population which is relatively low [17, 49]. In addition, restriction of ultra-processed foods is associated with lower Na intake (400–500% lower) and higher K intake (300–400% higher) in comparison with the typical industrialized and Western diets [50, 51].

We observed a moderate correlation between the PaleoDiet score and two MedDiet indices. Moreover, a stronger inverse association of the PaleoDiet with CVD was observed when adherence to the MedDiet was highest. This finding means that although the PaleoDiet is proxy of a healthy eating model, the MedDiet may exert even higher cardiovascular benefits. Contrary to the MedDiet, the PaleoDiet recommends a low consumption of legumes and whole cereals and grains, and a high consumption of lean meat (white or red meat). Several studies have suggested that legume consumption improves multiple cardiovascular risk factors (blood pressure, LDL concentrations in blood, body weight) and protects against type 2 diabetes, reducing glycated hemoglobin levels in diabetic patients and improving insulin sensitivity [52, 53]. In addition, the limitation of whole grains consumption in the PaleoDiet may hamper that key nutrients needs such as fiber, vitamins, minerals, lignans, and phytochemicals (phenolic acids, polyphenols, and phytosterol compounds) are met [54, 55]. These nutrients have been positively associated with longevity, and lower risk of obesity, type 2 diabetes, heart disease and colon cancer [54–57]. Finally, there is still some controversy about the cardiovascular effect of unprocessed red meat [58, 59], but several recent analyses [60–62] suggested a positive association between the consumption of unprocessed red meats and cardiovascular risk. Moreover, no evidence from these studies suggests any cardiometabolic benefits of unprocessed red meat consumption [59].

This study has some limitations. First, the SUN cohort participants did not actively choose a lifestyle according to a PaleoDiet, since we applied an a posteriori classification criterion using a self-reported semi-quantitative FFQ. A randomized intervention study with long-term follow-up will be needed to assess the effect of an increased adherence to the PaleoDiet, and also to other lifestyle factors associated with this diet, on cardiovascular risk. Second, we used a self-reported semi-quantitative FFQ, which is susceptible to non-differential measurement error, which would more likely underestimate the true association. However, the FFQ is considered as the most appropriate and practical approach to assess usual food consumption in large cohorts [22]. Third, our participants were young adults with low prevalence of cardiovascular risk factors and this explains the small number of observed cardiovascular events, especially among women. Further analyses in larger studies are needed to confirm our results and to explore potential interactions with variables such age, sex or other lifestyles such as physical activity. Fourth, the SUN cohort did not collect biomarkers of cardiovascular risk factors and therefore we were not able to identify potential mechanisms that could explain the inverse association between the PaleoDiet and the risk of CVD. Five, the FFQ was not specifically designed to collect data about the new NOVA classification of ultra-processed foods consumption. We could not include some items (cereal and energy bars, energy drinks, health and slimming products, and meat or vegetable nuggets) because the FFQ of the SUN project did not include these items. Therefore, there is the potential for some degree of misclassification of ultra-processed food consumption inherent in our methodology. However, our FFQ was previously validated and represents the main foods ingested by the studied population [23], including ultra-processed foods and this potential misclassification would be non-differential according to the status of participants at the end of the study. Six, we cannot rule out the existence of residual confounding, although we adjusted for the well-known risk factors of CVD in different multivariable models and the results were very similar regardless of the variables used for adjustment. Seven, another limitation is the difficulty of measuring the PaleoDiet, although we used a new index based on our previous comprehensive review [17]. Current adaptations of the PaleoDiet may vary from some archeological records [63] and, for example, although potato intake was not included in our score, some studies suggest that root vegetables with high starch content may be a component of Paleolithic diets [64]. Finally, the external validity of our results is limited since participants in our cohort were relatively young with high educational level and with low prevalence of cardiovascular risk factors, which limits the generalization of the results to the general population. However, lack of representativeness does not necessarily imply lack of validity, and the inclusion of highly educated participants in our cohort improves the quality of self-reported information and reduces the possible confounding by educational level and other socio-economic factors [22]. In addition, the characteristics of our cohort do not prevent from establishing associations that can be generalized to other groups, as long as similar biological mechanisms are plausible in these populations.

Strengths of our study are the high retention rate (91%), the blind confirmation of CVD events by a cardiologist, which minimizes the potential for misclassification, the prospective design, which limits the possibility of reverse causality, the ability to control for a large number of potential confounders and the long-term follow-up. Lastly, we conducted a wide array of sensitivity analyses to test the robustness of our results.

In conclusion, our results suggest that the PaleoDiet may decrease CVD risk in young adult participants from a Mediterranean country. This association could be explained by the synergistic effect of all the items of the PaleoDiet score although low consumption of ultra-processed foods, and high consumption of fruits and vegetables seem to be key components to reduce CVD risk. A slightly higher inverse association was also found when cereals and grains were not negatively scored in the PaleoDiet. Moreover, a stronger inverse association with the PaleoDiet was found as the level of adherence to the MedDiet increased. Further research with different populations and longer follow-up is needed to replicate these findings and to better clarify the health impact that restriction of typical Mediterranean foods, such as legumes and whole grains, may have on the prevention of CVD for the general population.

## Supplementary Information

Below is the link to the electronic supplementary material.

Supplementary file (DOCX 959 KB)

## Data Availability

This study uses data from the *Seguimiento Universidad de Navarra* (SUN) cohort. All data and materials as well as software application or custom code used during the current study shall be made available from the corresponding author on reasonable request.

## Abbreviations

BMI: Body mass index
CI: Confidence interval
CVD: Cardiovascular disease
FFQ: Food Frequency Questionnaire
HR: Hazard ratio
LDL: Low density lipoprotein
MDS: Mediterranean diet score
MEDAS: MEditerranean Diet Adherence Screener
METs: Metabolic equivalents of task
MedDiet: Mediterranean diet
MUFAs: Monounsaturated fatty acids
PaleoDiet: Paleolithic diet
PREDIMED: PREvención con DIeta MEDiterránea
P: Percentile
PUFAs: Polyunsaturated fatty acids
RR: Relative risk
SD: Standard deviation
SFAs: Saturated fatty acids
SUN: Seguimiento Universidad de Navarra
