# Supplementary material for: A score appraising Paleolithic diet and the risk of cardiovascular disease in a Mediterranean prospective cohort
Source: Eur J Nutr. 2021 Oct 21;61(2):957–71. doi: 10.1007/s00394-021-02696-9 (PMC8854325; doi:10.1007/s00394-021-02696-9)
Supplement: Supplementary file 1 — Supplementary file (DOCX 959 KB) [file 394_2021_2696_MOESM1_ESM.docx]

**Supplemental table 1.** Summary of the variables that compose each food group of the Paleolithic diet score.

|  | **PaleoDiet score** | |
| --- | --- | --- |
| **Dietary score food group** | **Variables** | **g per serving of FFQ*** |
| Fruits | Orange, grapefruit, tangerine  Banana    Apple pear  Strawberries    Peach, apricot, nectarine  Cherry, plum  Figs  Grapes    Mango, papaya  Kiwi  Cantaloupe    Watermelon | 150  100  150  60  150  150  150  150  150  100  225  225 |
| Vegetables | Chard, spinach  Cabbage, cauliflower, broccoli    Lettuce, endives, escarole  Raw tomato  Carrot, pumpkin  Green bean  Eggplant, courgettes, cucumber  Pepper  Asparagus    Gazpacho    Other vegetables | 250  250  250  150  250  250  250  250  250  250  250 |
| Fishes | White fish: whiting, hake, sea bream, halibut, sole    Blue fish: sardines, tuna, bonito, mackerel, salmon  Cod  Salted / smoked fish  Oysters, clams, mussels  Prawns, crayfish    Octopus, squid, baby squid | 150  150  150  50  60  100  150 |
| Unprocessed meats | Chicken / Turkey with skin  Chicken / Turkey without skin    Pork    Lamb    Rabbit, hare  Liver  Viscera  Serrano ham  Bacon  Beef  Cow | 150  150  150  150  150  100  100  50  50  150 |
| Tree nuts | Almonds, peanuts, hazelnuts, walnuts | 50 |
| Eggs | Egg | 60 |

*Baseline FFQ available in [*https://www.unav.edu/web/departamento-de-medicina-preventiva-y-salud-publica/proyecto-sun/informacion-para-investigadores*](https://www.unav.edu/web/departamento-de-medicina-preventiva-y-salud-publica/proyecto-sun/informacion-para-investigadores)

**Supplemental table 1.** Continuation.

|  | **PaleoDiet score** | |
| --- | --- | --- |
| **Dietary score food group** | **Variables** | **g per serving of FFQ*** |
| Cereals and grains | White bread  Black bread  Rice  Pasta | 60  60  60  60 |
| Dairy products | Whole milk  Semi-skimmed milk  Skimmed milk  Condensed milk  Cream or milk cream  Milk shake  Whole yogurt    Non-fat yogurt  Curd  Cheese in portions  Other cheese: cured or semi-cured  Fresh cheese | 200  200  200  15  100  200  125  125  100  30  50  50 |
| Legumes | Lentils  Chickpeas  Bean  Green peas | 60  60  60  60 |
| Ultra-processed food | Petit Suisse    Custard, pudding  Ice cream  Ham cooked  Sausages  Pate and foie-gras  Blood sausage  Burger  Spicy sausage/meatballs  Potato chips  Breakfast cereals  Pizza (including pre-prepared pies)  Margarine  Fried out of home  Frying with margarine  Saccharin  Cookies  Chocolate cookies  Muffins  Doughnuts  Croissant or other non-handmade pastries  Cakes  Churros, batons  Chocolate and candies  Nougat    Marzipan, tea paste, shortbread  Carbonated drinks  Artificially sugared beverages  Fruit drinks  Milk shakes  Soups and creams of envelope  Croquettes  Mayonnaise  Alcoholic drinks produced by fermentation followed by distillation such as whisky, gin, and rum | 100  200  100  50  50  25  50  100  50  150  30  200  10  10  10  1  50  50  45  50  50  50  100  30  50  90  200  200  200  250  135  10  50 |
| Culinary ingredients | Added salt  Added sugar  Butter  Lard  Sunflower oil  Corn oil  Other refined vegetable oils | 1  10  10  10  10  10  10 |

*baseline FFQ available in [*https://www.unav.edu/web/departamento-de-medicina-preventiva-y-salud-publica/proyecto-sun/informacion-para-investigadores*](https://www.unav.edu/web/departamento-de-medicina-preventiva-y-salud-publica/proyecto-sun/informacion-para-investigadores)

**Supplemental Table 2**. The 14-point Mediterranean Diet Adherence Screener (MEDAS) and 9-point Mediterranean Diet Adherence by Antonia Trichopoulou.

| **14-point Mediterranean Diet Adherence Screener (1)** | **Score**  **(criteria for 1 point)** | **9-point Mediterranean Diet Adherence (2)** | **Score**  **(criteria for 1 point)** |
| --- | --- | --- | --- |
| *1. Do you use olive oil as the principal source of fat for cooking?* | Yes | *1. Vegetables* | ≥ median |
| *2. How much olive oil do you consume per day (including that used in frying, salads, meals eaten away from home, etc.)?* | 4 or more tablespoons | *2. Legumes* | ≥ median |
| *3. How many servings of vegetables do you consume per day? Count garnish and side servings as 1/2 point; a full serving is 200 g* | ≥2 | *3. Fruits and nuts* | ≥ median |
| *4. How many pieces of fruit (including fresh-squeezed juice) do you consume per day?* | ≥3 | *4. Cereals* | ≥ median |
| *5. How many servings of red meat, hamburger, or sausages do you consume per day? A full serving is 100–150 g* | < 1 | *5. Fish* | ≥ median |
| *6. How many servings (12 g) of butter, margarine, or cream do you consume per day?* | < 1 | *6. Monounsaturated/saturated* | ≥ median |
| *7. How many carbonated and/or sugar-sweetened beverages do you consume per day?* | < 1 | *7. Meat/meat products* | < median |
| *8. Do you drink wine? How much do you consume per week?* | ≥7 glasses | *8. Dairy products* | < median |
| *9. How many servings (150 g) of pulses do you consume per week?* | ≥3 | *9. Ethanol (men 10-50g/d and women 5-25g/d)* | Yes |
| *10. How many servings of fish/seafood do you consume per week? (100–150 g of fish, 4–5 pieces or 200 g of seafood)* | ≥3 |  |  |
| *11. How many times per week do you consume commercial sweets or pastries (not homemade), such as cakes, cookies, biscuits, or custard?* | < 2 |  |  |
| *12. How many times do you consume nuts per week? (1 serving = 30 g)* | ≥3 |  |  |
| *13. Do you prefer to eat chicken, turkey or rabbit instead of beef, pork, hamburgers, or sausages?* | Yes |  |  |
| *14. How many times per week do you consume boiled vegetables, pasta, rice, or other dishes with a sauce of tomato, garlic, onion, or leeks sautéed in olive oil?* | ≥2 |  |  |

For both scores, 0 points if these criteria are not met.

(1) Schröder H, Fitó M, Estruch R, Martínez‐González MA, Corella D, Salas‐Salvadó J, Lamuela‐Raventós R, Ros E, Salaverría I, Fiol M, et al. A Short Screener Is Valid for Assessing Mediterranean Diet Adherence among Older Spanish Men and Women. J Nutr. 2011;141:1140–5.

(2) Trichopoulou A, Costacou T, Bamia C, Trichopoulos D. Adherence to a Mediterranean diet and survival in a Greek population. N Engl J Med. 2003;348:2599–608.

**Supplemental Table 3.** Hazard ratios and confidence intervals according to joint classification by combined exposures to the PaleoDiet and the Mediterranean Diet Adherence Screener (MEDAS)^1^.

|  |  | Paleolithic diet score (min-max) | | |
| --- | --- | --- | --- | --- |
|  |  | **Q1** | **Q2-Q4** | **Q5** |
|  |  | (16-28) | (29-36) | (37-50) |
| Mediterranean Diet Adherence Screener (MEDAS) (min-max) | **≤median** (0-6) | 1 (Ref.) | **0.62 (0.39-0.97)** | **0.27 (0.11-0.68)** |
|  | **>median** (7-13) | 0.79 (0.35-1.82) | **0.57 (0.34-0.95)** | **0.51 (0.28-0.92)** |
|  |  |  |  |  |

Abbreviations: HR, Hazard Ratio; CI, Confidence Interval; Q, Quintile; Ref., Reference.

^1^ Mediterranean Diet Adherence Screener (MEDAS) was assessed using in the Prevención con Dieta Mediterránea (PREDIMED) trial [32].

Hazard ratio adjusted for age (10 groups) as the underlying variable of time, sex, year entering the cohort (1999-2001, 2002-2004, 2005-2007, 2008-2010, 2011-2014, 2015-2017), total energy intake (continuous), alcohol intake (teetotaler, >0-5 g/d in females and >0-10 g/d in males, >5-25 g/d in females and >10-50 g/d in males, >25 g/d in females and >50 g/d in males), smoking status (non-smoker, ex-smoker, current smoker), BMI (continuous), physical activity (METs-h/week as continuous), prevalent hypertension, hypertriglyceridemia, hypercholesterolemia, diabetes, cancer, depression and family history of CVD (yes/no), education level (graduate, master, doctorate) and smoking-pack-years (continuous), squared BMI, napping (yes/no), watching television (h/day), sitting time (h/week), snacking between meals (yes/no) and following special diets (yes/no).

**Supplemental Table 4**. Sensitivity analyses: Adjusted Hazard Ratios (HRs) and 95 % confidence intervals (CI) of incident Cardiovascular Disease by quintiles of PaleoDiet score (Q5 vs. Q1).

|  | N | CVD events | HR (95% CI) | *P*-trend^1^ |
| --- | --- | --- | --- | --- |
|  |  |  |  |  |
| Overall | 18,214 | 165 | 0.45 (0.27, 0.76) | 0.008 |
| Including non-confirmed CVD events | 18,214 | 209 | 0.47 (0.29, 0.76) | 0.003 |
| Excluding participants with total energy intake^2^ <P1 and >P99 | 19,772 | 172 | 0.57 (0.34, 0.96) | 0.010 |
| Excluding participants with special diet at baseline | 16,317 | 143 | 0.43 (0.25, 0.74) | 0.001 |
| Excluding participants <40 years | 7,268 | 149 | 0.35 (0.20, 0.63) | <0.001 |
| Excluding participants with chronic aspirin intake | 17,595 | 155 | 0.41 (0.22, 0.76) | 0.004 |
| Excluding those with hypertension at baseline | 16,952 | 115 | 0.49 (0.25, 0.97) | 0.010 |
| Excluding those with aneurysm of the aorta, heart failure, atrial fibrillation, pulmonary embolism, peripheral venous thrombosis and intermittent claudication | 17,657 | 138 | 0.46 (0.24, 0.86) | 0.006 |
|  |  |  |  |  |

^1^ Test for lineal trend calculated for the 5 quintiles.

^2^ Total energy intake <P1 (<1073 kcal) and >P99 (>3777 kcal).

Hazard ratio and 95% confident intervals adjusted for sex, stratified for age deciles and year entering the cohort (1999-2001, 2002-2004, 2005-2007, 2008-2010, 2011-2014, 2015-2017), total energy intake (continuous), alcohol intake (teetotaler, >0-5 g/d in females and >0-10 g/d in males, >5-25 g/d in females and >10-50 g/d in males, >25 g/d in females and >50 g/d in males), smoking status (non-smoker, ex-smoker, current smoker), BMI (continuous), physical activity (METs-h/week ), prevalent hypertension, hypertriglyceridemia, hypercholesterolemia, diabetes, cancer, depression and family history of CVD (yes/no), education level (graduate, master, doctorate) and smoking-pack-years (continuous), squared BMI, napping (yes/no), watching television (h/ day), sitting time (h/ week), snacking between meals (yes/no) and following special diets (yes/no).

Abbreviations: CVD, Cardiovascular Disease; P, Percentile.

A)


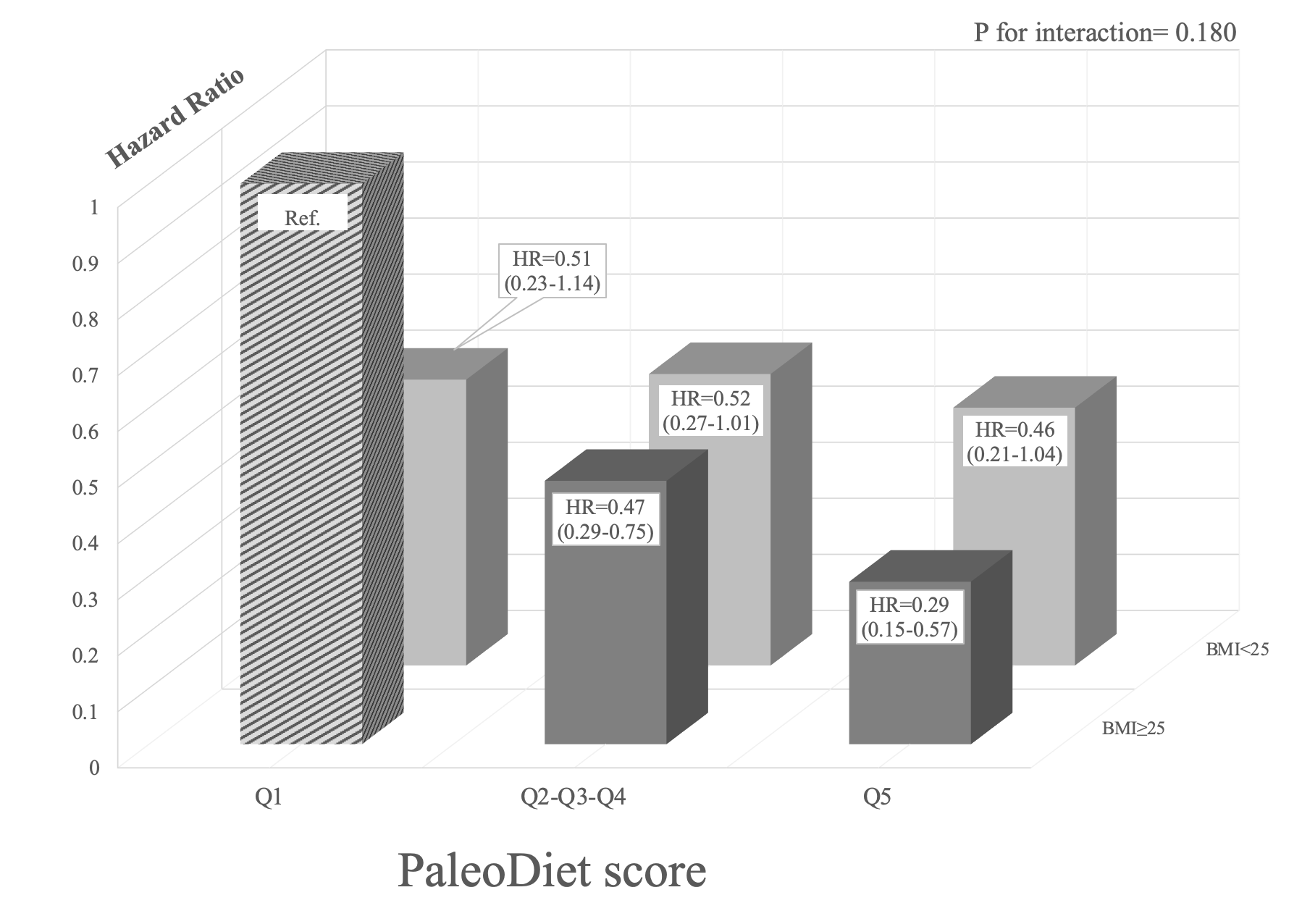


B)


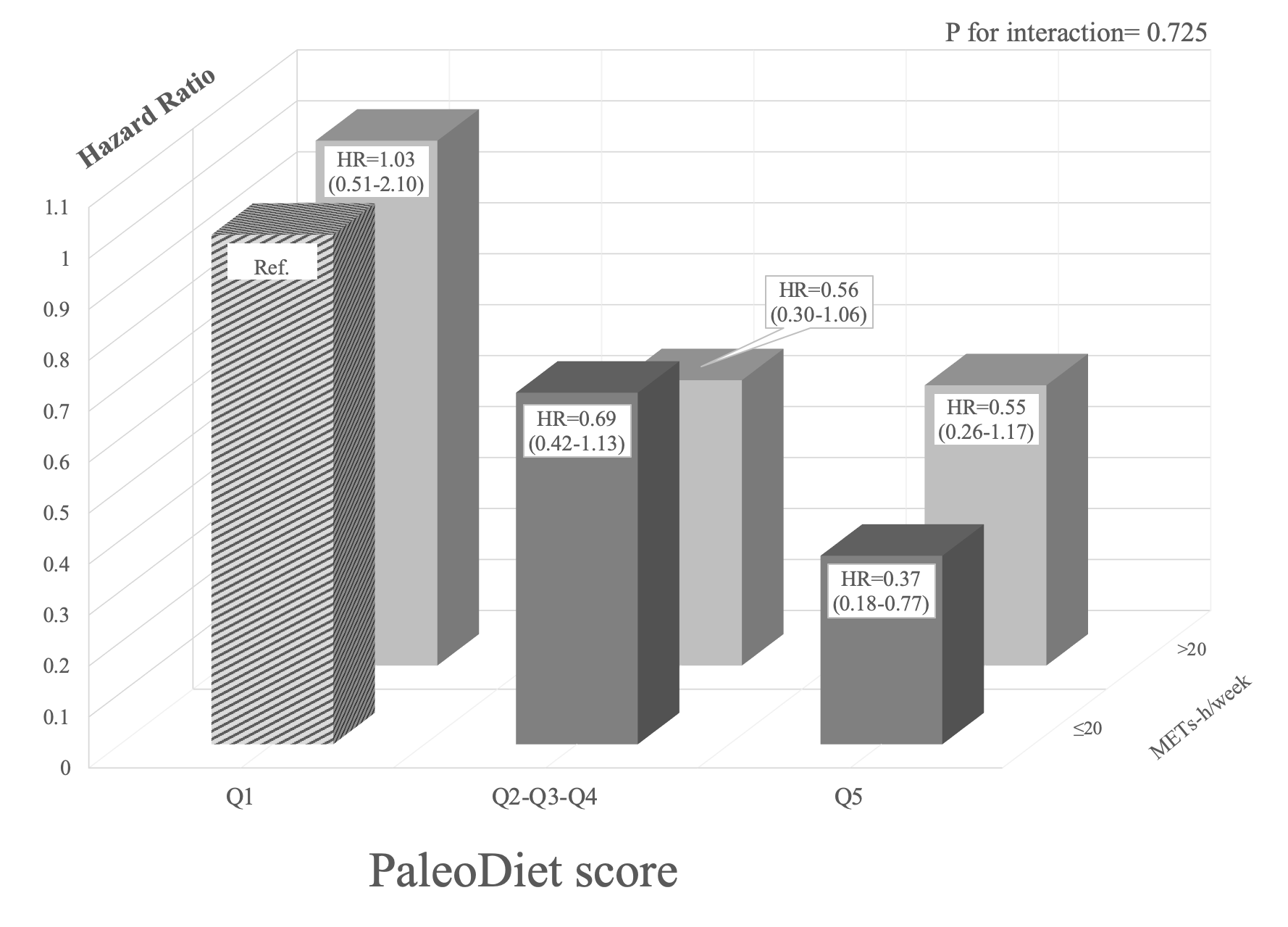


C)


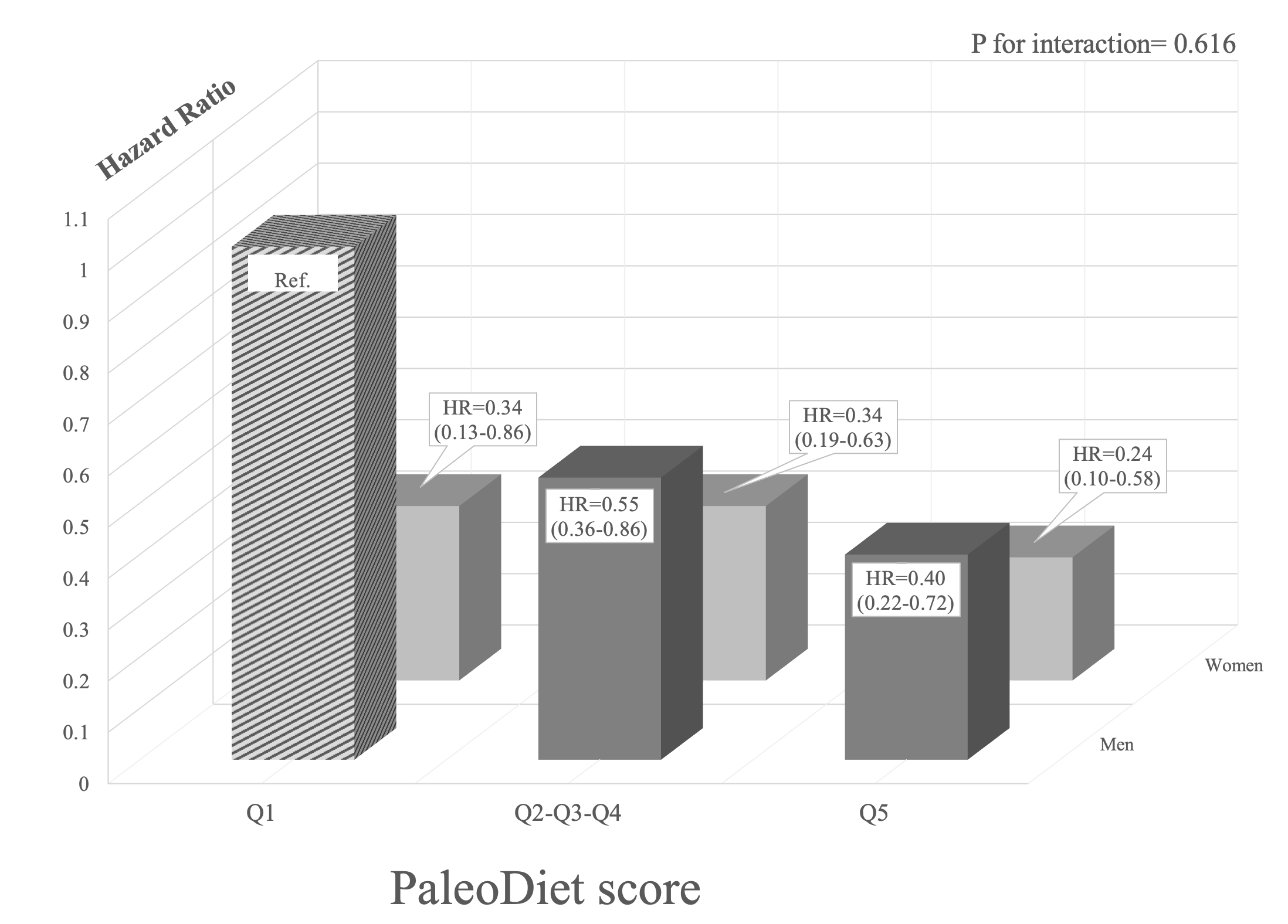


**Supplemental Figure 1**. Hazard ratios of combined effects of PaleoDiet (assessed with the PaleoDiet score) and (A) BMI (<25; ≥25 kg/m^2^), (B) physical activity (≤20 METs-h/week; >20 METs-h/week) and (C) sex. Hazard ratio and 95% confident intervals adjusted for sex, stratified for age deciles and year entering the cohort (1999-2001, 2002-2004, 2005-2007, 2008-2010, 2011-2014, 2015-2017), total energy intake (continuous), alcohol intake (teetotaler, >0-5 g/d in females and >0-10 g/d in males, >5-25 g/d in females and >10-50 g/d in males, >25 g/d in females and >50 g/d in males), smoking status (non-smoker, ex-smoker, current smoker), BMI (continuous), physical activity (METs-h/week), prevalent hypertension, hypertriglyceridemia, hypercholesterolemia, diabetes, cancer, depression and family history of CVD (yes/no), education level (graduate, master, doctorate) and smoking-pack-years (continuous), squared BMI, napping (yes/no), watching television (h/ day), sitting time (h/ week), snacking between meals (yes/no) and following special diets (yes/no).
